# Interaction Between the Intestinal Microbial Community and Transcriptome Profile in Common Carp (*Cyprinus carpio* L.)

**DOI:** 10.3389/fmicb.2021.659602

**Published:** 2021-05-28

**Authors:** Shengyan Su, Xiaojun Jing, Chengfeng Zhang, Yiran Hou, Zhixun Li, Xingli Yang, Xiaolin Zhou, Pao Xu, Yongkai Tang, Jian Zhu

**Affiliations:** ^1^Key Laboratory of Genetic Breeding and Aquaculture Biology of Freshwater Fishes, Ministry of Agriculture, Freshwater Fisheries Research Center, Chinese Academy of Fishery Sciences, Wuxi, China; ^2^Wuxi Fisheries College, Nanjing Agricultural University, Wuxi, China; ^3^College of Fisheries, Huazhong Agricultural University, Wuhan, China; ^4^Henan Academy of Fishery Sciences, Zhengzhou, China

**Keywords:** transcriptome, gastrointestinal tract, 6S DNA, Huanghe carp new strain, interaction, transcriptome profile

## Abstract

In a previous study, we found that the growth performance of the new strain of Huanghe carp is related to gene expression and bacterial community in the gut. In order to better understand the relationship between the gene expression level and bacterial abundance in the gut, we studied the growth performance, gut bacterial structure, and transcriptome profile in the 4th generation of the new carp strain (selection group) at harvesting time, and compared them with the control line (traditional Huanghe carp). Body weight, depth, width, and length increased 14.58, 7.14, 5.04, and 5.07%, respectively. The gut microbiome of the selection group also exhibited significantly higher species diversity parameters (Shannon, Simpson, and chao1). Both PCA and phylogenetic analyses divided all gut samples into two parts: control and selection group. *Aeromonas* was the dominant taxon in the control group, followed by *Firmicutes* and *Roseomonas*; in the selection group, *Roseomonas* was the dominant taxon, followed by *Firmicutes* and then *Aeromonas*. Among the 249 significantly differentially expressed genes, 194 were downregulated and 55 were upregulated. Functional GO annotation produced 13 terms in the biological process, 8 in the cellular component, and 7 in the molecular function categories. KEGG annotation indicated that most of these genes were associated with the immune-related pathways. A total of 2,892 pairs of genes (245) and baceterial genera (256) were analyzed using Pearson’s correlation analysis. Most of the identified associations were mapped to the immune system, bacterial community, and cell differentiation categories. The top-10 bacterial genera identified by these analyses were *Methylocystis*, *Ohtaekwangia*, *Roseomonas*, *Shewanella*, *Lutispora*, *GpVI*, *Desulfovibrio*, *Candidatus_Berkiella*, *Bordetella*, and *Azorhizobium*. Genes paired with bacteria flora were divided into four functional categories: immune, growth, adipocyte differentiation, and nerve regulation. These genes may be related to the comparatively fast growth and high muscle polyunsaturated fatty acid content of the Huanghe carp new strain. Meanwhile, nerve regulation-related genes may be a reflection of the microbiota-gut-brain axis. These results illustrate that gut bacterial community structure is associated with the growth performance and gene expression in the Huanghe carp new strain.

## Introduction

In recent years, the influence of gut microbes on the host’s production performance has received extensive attention. In mammals, gut microbiota affect the lipid metabolism mainly via short-chain fatty acids, secondary bile acids, trimethylamine, and bacterially sourced proinflammatory factors, such as lipopolysaccharides (Schoeler and Caesar, 2019). This process mainly involves the bile acid signal transduction pathway, mainly relying on the bile acid receptor farnesoid X receptor (FXR), the G-protein-coupled bile acid receptor (Gpbar/TGR5), the short-chain fatty acid signal pathway, and enteroendocrine cell function (Yu et al., 2019). Through structural model equation analysis, it was found that the changes of eukaryotic microbial flora in shrimp intestines are positively correlated with digestive enzyme activities, and that the synergy between them promotes the rapid growth of shrimp (Dai et al., 2017). In sheep fed with tannins for 122 days, C18:0/C18:1t11 ratio increased (via transformation of C18:2c9-c12 into C18:2c9-t11 and C18:1t11 into C18:0) along with the abundance of *Vibrio cellulolyticus butyricum* in the rumen, which suggests a relationship between the gastrointestinal flora and transformation of fatty acid components (Vasta et al., 2010).

The production performance of the host can be regulated by adjusting the structure of intestinal microbial flora, which in turn is mainly affected by genetics and feed. Intestinal microbes are also able to affect the host’s gene expression and methylation levels (Levy et al., 2015). A comparative study of the structure of the intestinal microbial flora of transgenic carp [Based on the technique of microinjection, recombinant recombinant grass carp (*Ctenopharyngodon idellus*) growth hormore gene (*gcGH*) has been transferred into fish eggs] and wild carp found that *Proteobacteria*, *Fusobacteria*, *Bacteroides*, and *Firmicutes* exhibited significant differences in abundance, and that carbohydrate significantly increased in the transgenic carp. However, lipid metabolism and the abundance of Bacteroides and Firmicutes were significantly lower than in the wild carp, which suggests that difference in intestinal microbes is significantly correlated to the growth performance of transgenic carps (Li et al., 2013). Research has been conducted into how this intestinal microbial flora associated with germplasm resources forms a stable structure. Wang et al. (2014) found that Proteus, Bacteroides, Verrucous bacteria, Flat bacteria, and Hardwall bacteria were the dominant microbial flora in juvenile grass carp. Besides this, the structure of the flora differed among developmental stages; e.g., *proteobacteria* was the dominant taxon in the fertilized egg stage (Wang et al., 2015). In a study of the structure of intestinal microflora during different growth stages of *Litopenaeus vannamei* culture, it was found that Proteobacteria, Bacteroides, and Actinomycetes were observed in all growth stages, but dominant species varied among the growth stages: Comamonadaceae of Betaproteobacteria at 2 weeks and 1 month, Flavobacteria at 2 months, and Vibrio at 3 months (Huang et al., 2016). Through the analysis of water quality, bottom sediment (mud), and the intestinal microbial flora structure of *Eriocheir sinensis* in different growth and development stages, it was found that Proteobacteria, Firmicutes, Soft-walled, and Bacteroides are the endogenous intestinal flora. The influence of the environment on the intestinal microflora becomes smaller with time (Wang et al., 2019). With regards to the interaction mechanism between the gut microbial flora and host gene expression, studies have found that about 10% of the host transcriptome is regulated by microorganisms, mainly including genes in immune, cell proliferation, and metabolic functions. The influence of microorganisms on host gene expression is highly site-specific, and each cell fraction is enriched with specific transcriptional regulators (Sommer et al., 2015). This influence is mainly achieved through the gut-brain axis and the gut-hepatic axis. The former involves hormones, immunity, and nerve signal transduction. This interaction can affect dietary behavior, digestive process, immune function, and other physiological phenomena (Butt and Volkoff, 2019). The gut-hepatic axis plays a key role in the establishment of the host’s intestinal microbes in the early development. Early consumption of starter feed for 5 weeks promoted the development of lambs’ rumen, and increased the activities of rumen microbial amylase and carboxymethyl cellulase. The rumen flora of breastfed sheep is more diverse than that of colostrum-fed sheep. The mechanism may involve the stimulation of rumen ketogenesis and butyrate metabolism by genes hydroxymethylglutaryl-CoA lyase, mitochondrial (HMGCL), and 3-hydroxy-3-methylglutaryl-CoA synthase 2 mitochondrial (HMGCS2), as well as by the change of fermentation type caused by rumen microbiota (Wang et al., 2016). In this microbe-gene expression pattern of the gut-hepatic axis, another important research direction is the change of circadian rhythm. Studies have shown that under the effect of continuous light and dark cycles, compared with conventionally reared mice, sterile mice on a low-fat or high-fat diet showed obvious damage to central and liver circadian rhythm gene expression, and did not gain weight. In the intestinal flora of routinely reared mice, it was found that the daily changes of microbial structure and function vary with diet composition. Under low-fat or high-fat feeding regime, short-chain fatty acids, rather than specific microbial metabolites induced by hydrogen sulfide, directly regulate the expression of circadian clock genes in liver cells (Leone et al., 2015). The activity of the microbial flora over the course of a day can also cause changes in the host’s circadian rhythm, epigenetics, and metabolites. When the rhythm of the homeostasis of the microbial community is disrupted, the normal chromatin and gene expression levels of the host will vary, and the new mechanism of gene expression in the genome’s gut-hepatic axis will be activated (Thaiss et al., 2016).

The interaction between the above-mentioned intestinal flora and the host is mainly achieved through the remote-control mode of the brain and liver (Fu and Cui, 2017; Martin et al., 2018). When using intestinal microbes from healthy people to treat colonic epithelial cells, Richards et al. (2019) found that intestinal flora can regulate the expression of host genes through chromatin accessibility and transcription factor binding induced by exposure to gut microbiota (Richards et al., 2019). In addition, the intestinal flora can also affect intestinal morphology. Using *Drosophila* as the research object, the comparison of gene expression between wild-type and immunodeficient groups showed that 53% of upregulated genes exerted their effects through immunodeficiency (IMD) signal pathways, due to the way the intestinal flora affects the intestinal morphology by affecting the cell spacing, the renewal rate of epithelial cells, and the composition of different cell types in the epithelium (Broderick et al., 2014). In terms of regulatory pathways, by knocking out the myeloid differentiation primary response 88 (MyD88) gene, it was found that the host gene expression in the small intestine and part of the colon was regulated by intestinal microbes but few microbiota regulated genes required *MyD88* signaling pathway (Larsson et al., 2012). Especially strongly affected was the expression of antibacterial genes regenerating islet-derived protein 3 beta (Reg3β) and regenerating islet-derived protein 3 gamma (Reg3γ) in colonic epithelial cells. Therefore, this study relied on transcriptome sequencing to understand the way in which the structure of the intestinal microbiota of the new Huanghe carp strain affects its growth performance by regulating the expression of intestinal genes. The goal was to provide a reference for suitable breeding methods of the new Huanghe carp strain.

## Materials and Methods

### Experimental Fish and Rearing Facility

Huanghe carp new strain (Shengyan et al., 2018) fingerlings were obtained from the Freshwater Fisheries Research Centre of Chinese Academy of Fishery Sciences (Wuxi, China). The control and selection groups (1,500 specimens each, initial average body weight 3.72 g on June 7, 2018) were reared in two separate ponds at the Henan Academy of Fishery Science. The geographic positions of the ponds were measured with a portable GPS. Fish were fed commercial feed at a daily rate of 3% of their body weight (adjusted weekly) three times a day (08:00, 12:00, and 16:00) by the same person in the same manner. During the trial, from June to October, water parameters were observed for 10 time points (each point has three points along the diagonal with equal distance in each pond; Supplementary Table 1). In that period, water temperature ranged from 15 to 35°C. All experimental protocols were carried out according to the Guide for the Care and Use of Animals of China, and were approved by the Animal Care and Use Committee of the Chinese Academy of Fishery Sciences.

After 5 months, 366 individuals were randomly sampled (control group 180 individuals, selection group 186 individuals). Body weights (hBwt) were measured using electronic scales (accuracy: 0.1 g), whereas body lengths (hBlen), widths (hBwid), and depths (hDep) were measured using a plastic ruler (accuracy: 0.1 cm). Student’s *t*-test was used to examine differences between the selection and control groups (with 0.05 selected as the significance threshold).

### Gut Sampling

After 5 months, three fishes (starved for 24 h) were randomly sampled from each pond (DD4 refers to samples from the control group, whereas SS4 refers to samples from the selection group; Supplementary Table 2), transported to the laboratory alive, anesthetized using 100 mg/l tricaine methanesulfonate (MS-222), and dissected under aseptic conditions. The intestines were isolated from the body cavity using a sterile pair of scissors and forceps, divided into foregut, midgut, and hindgut segments (to make sure that we sampled gut contents from all representative gut segments), and the contents of each segment were harvested into sterile 2 ml collection tubes by squeezing with forceps (Li et al., 2015). After that, the samples were immediately frozen in liquid nitrogen and later stored at −80°C until further analyses.

### DNA Extraction, PCR Amplification, Purification and Pyrosequencing

Cetrimethylammonium bromide (CTAB) and sodium dodecyl sulfate (SDS) were used to extract the total genomic DNA, the concentration and purity of which were determined by electrophoresis on 1% agarose gels stained with ethidium bromide. After that, DNA samples (1 ng/μL) were stored at −80°C.

The V4 and V3 hypervariable region of the bacterial *16S rRNA* gene (400–450 bp) was amplified using the universal PCR primers 515F [5′-GTGCCAGCMGCCGCGGTAA-3′ (Li et al., 2015)] and 806R [5′-GGACTACHVGGGTWTCTAAT-3′ (Apprill et al., 2015)]. PCR reactions were performed using Phusion^®^, High-Fidelity PCR Master Mix (New England Biolabs). A total reaction volume of 50 μL contained 1 μL DNA template (∼5 ng), 2.5 μL of each forward and reverse primer (10 pmol each), 25 μL Phusion^®^, High-Fidelity PCR Master Mix (New England Biolabs), and 19 μL nuclease-free water. Experiments were conducted in triplicate. The amplification settings were: initial denaturing at 98°C for 30 s and then 35 cycles of 10 s at 98°C (denaturing), 30 s at 65°C (annealing), and 30 s at 72°C (extension), and the last step was an extension for 10 min at 72°C. PCR products were checked on 2% agarose gels and purified with Qiagen Gel Extraction Kit (Qiagen, Germany).

TruSeq^®^, DNA PCR-Free Sample Preparation Kit (Illumina, United States) was used to generate the sequencing libraries. Sequencing was carried out on the Illumina MiSeq platform with 200 bp paired-end reads.

### Statistical and Bioinformatics Analysis

Firstly, MUTHOR (Li et al., 2015) was used to identify the operational taxonomic units (OTU), and conduct taxonomic and community composition evaluation. FLASH V1.2.7 (Magoč and Salzberg, 2011) was used to merge Paired-end reads. High-quality clean tags obtained from raw tags were subjected to quality control using QIIME V1.7.0 (Caporaso et al., 2010) and effective tags were also obtained by removing chimeric sequences using the UCHIME algorithm (Edgar et al., 2011). All sequences with ≥97% similarity were assigned to the same OTUs using Uparse software v7.0.1001 (Edgar, 2013). Each OTU was screened for further annotation using GreenGene Database (Madeira et al., 2019) on the basis of the Ribosomal Database Project (RDP) classifier 2.2 algorithm (Cole et al., 2009). Principal Coordinate Analysis (PCoA) was performed and results were visualized using WGCNA (Langfelder and Horvath, 2008), stat, and ggplot2 (Wickham, 2015) packages in R 2.15.3. The alpha diversity parameters (observed-species, Chao1, Shannon, and Simpson) and weighted UniFrac beta-diversity metric were calculated using QIIME 1.7.0 (Caporaso et al., 2010). The heatmap figure, Venn diagrams, and species rank abundance distribution curve were generated using R 2.15.3^1^. Non-metric multidimensional scaling (NMDS) was used to visualize the pairwise UniFrac distances among samples. Bernoulli correction tests were used to test the significance of differences.

### RNA Isolation, cDNA Library Construction and Illumina Sequencing

Total RNA extraction, cDNA libraries building, transcriptome sequencing, assembly, and annotation were conducted by the Decode Genomics Biotech Co., Ltd. (Nanjing, China). In total, three specimens from the Selected group and three specimens from the Control group were used for the transcriptome analyses (CT refers to samples from the control group, and ST refers to samples from the selection group) (Supplementary Table 2). Equal amounts of foregut, midgut, and hindgut segments belonging to the same specimen (see section “Gut Sampling”) were pooled together and RNA was extracted using TRIzol^®^, (Invitrogen, United States). After RNA integrity and concentration assessment using an Agilent Bioanalyzer 2100 system (Agilent Technologies, Santa Clara, CA, United States), cDNA libraries were structured using NEBNext Ultra^TM^ RNA Library Prep Kit for Illumina (NEB, United States) and purified using the AMPure XP system (Beckman Coulter, Beverly, MA, United States). The library preparations were sequenced on an Illumina Hiseq 2500 platform and paired-end reads were generated.

### Transcriptome Assembly, Annotation, Ontology, and Differential Gene Expression

Clean reads were generated from raw reads using WipeAadpter.pl and Fastq_filter (Biomarker Technologies, Beijing, China) scripts by removing adaptor-only reads, reads containing poly-N stretches (>5% total N), and low-quality reads (Q20-value ≤ 20). The clean reads for each sample were mapped to the reference common carp genome (Xu et al., 2014) using the Bowtie2 algorithm (Langmead et al., 2009) in TopHat2 (Kim et al., 2013). The FPKM (fragments per kilobase of transcripts per million fragments mapped) values were used to measure the expression level of each sample’s transcripts or genes (Mortazavi et al., 2008), and differentially expressed genes (DEGs) between the two groups (selection and control line) were explored using EBseq (Leng et al., 2013), with statistical significance assessed using the Benjamini-Hochberg procedure (Benjamini and Hochberg, 1995). Genes were defined as differentially expressed when they conformed to the parameters: | fold change| ≥ 2 and FDR < 0.01. Genes were annotated by querying against GO (Ashburner et al., 2000) and KEGG (Kanehisa and Goto, 2000) databases using BLASTx (Altschul et al., 1997). KEGG Orthology results were then obtained using KOBAS 2.0 (Xie et al., 2011). Pathway enrichment analysis was conducted using the KEGG database as described before (Chen et al., 2015).

## Results

### Growth Performance of Selection and Control Lines

**FIGURE 1:**
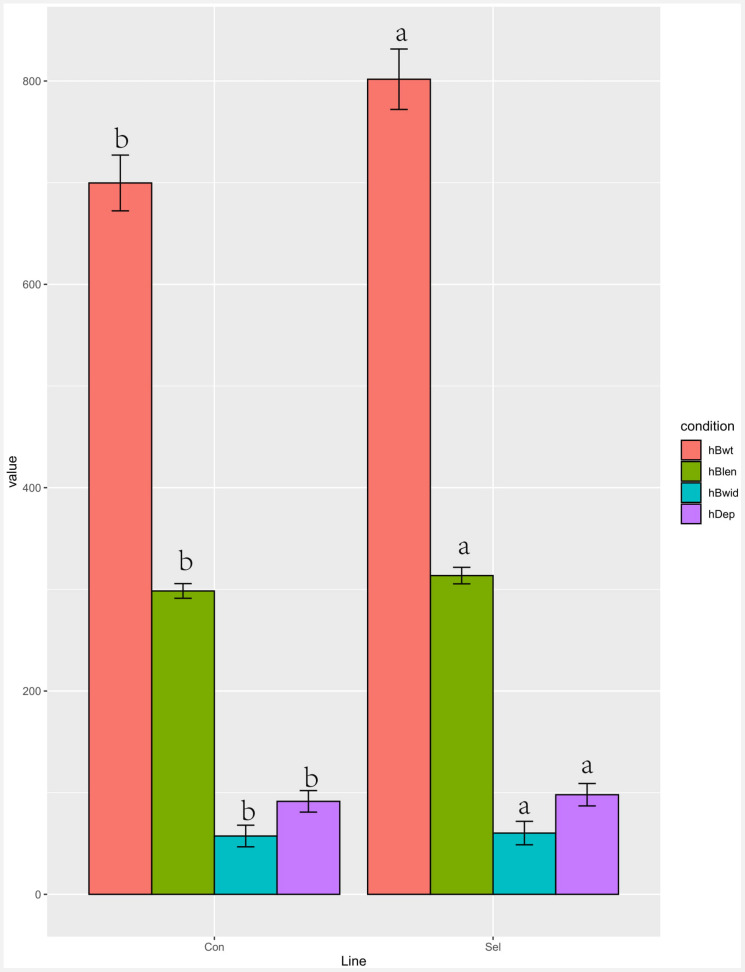
Growth performance comparison of the selection (Huanghe carp new strain 4th generation) and control lines. Different characters in the same color indicate a significant difference for a given growth parameter. Body weight is hBwt, length is hBlen, width is hBwid, and depth is hDep. *T*-test was used to examine the significance of differences between the selection and control groups (P di0.05).

Four growth performance parameters (body weight, length, width, and depth) were measured in the 4th generation of Huanghe carp new strain (Figure 1). All parameters were significantly improved in the selection line compared to the control lines at harvesting time: weight 14.58%, depth 7.14%, width 5.04%, and length 5.07%.

### Gut Bacterial Communities of Selection and Control Lines

**FIGURE 2:**
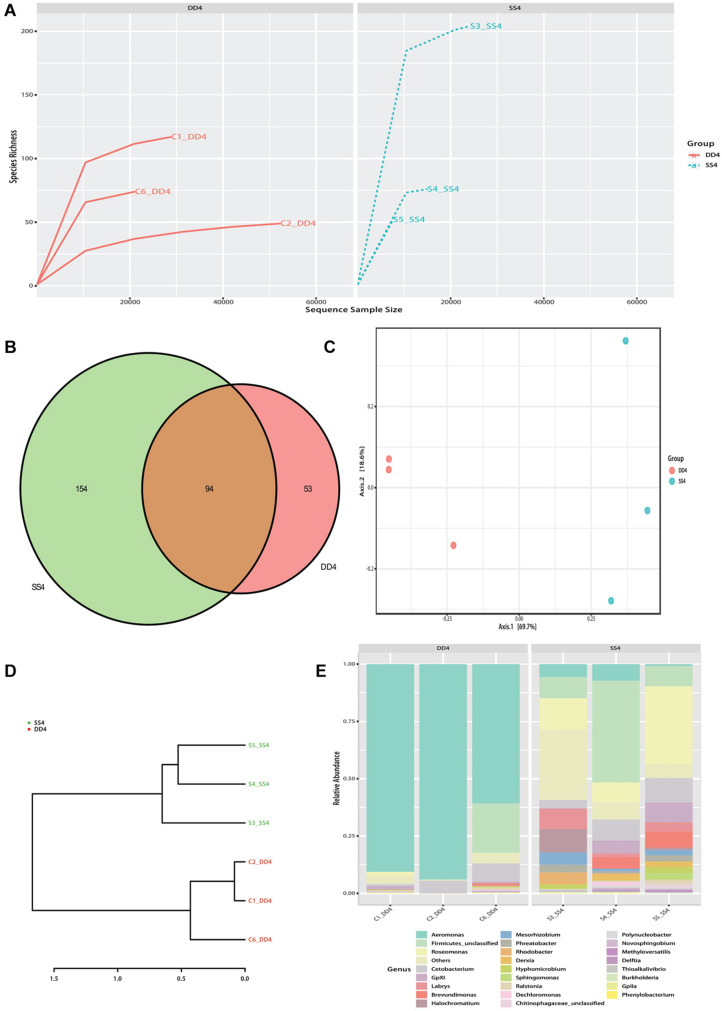
Bacterial composition analysis of the selection (Huanghe carp new strain 4th generation) and control lines. **(A)** Rank abundance curves for all samples’ gut bacteria communities. **(B)** Venn diagram showing gut bacteria of selection and control lines. **(C)** PCA plot of gut bacterial communities between the selection and control lines. **(D)** UPGMA tree based on weighted UniFrac distances. **(E)** Bar chart of relative bacterial abundance at genus level in selection and control lines. DD4 refers to the control group, and SS4 refers to the selection group.

An average of 68,252.5 raw tags and 64,837.3 effective tags per sample (95.23% of the raw tags) were obtained. For all valid tags, Q30 accounted for 93.15% and GC content was 52.72% (Supplementary Table 3). OTU richness of control samples ranged from 149 to 374, while the richness of selection samples ranged from 156 to 530. In total, 1,141 OTUs were isolated from all samples (Supplementary Table 4). The mean OTU richness was higher in the selection group (322.80 ± 67.87) than in the control group (303.00 ± 53.00). All samples’ rarefaction curves for the bacterial community reached an asymptote, which means that sufficient reads were examined to conduct downstream analyses (Figure 2A). Between selection and control groups, 94 shared bacterial taxa were identified (Figure 2B).

In order to better understand the bacterial diversity difference of microbial communities in these two groups, the number of observed species, along with the Shannon, Simpson, and chao1 indices, were calculated from the OTUs (Table 1). With regards to these four diversity parameters, the selection group exhibited a significantly higher difference from the control group. Both PCA analysis and phylogenetic tree divided all samples into two parts: control and selection group (Figures 2C,D). The analysis of bacterial composition (relative abundance) at the genus level indicates that *Aeromonas* was the dominant taxon in the control group, followed by *Firmicutes* and *Roseomonas* (Figure 2E). In the selection group, *Roseomonas* was the dominant taxon, followed by *Firmicutes* and then *Aeromonas*.

**TABLE 1:** Alpha_diversity.

	**Observed_species**	**Shannon**	**Simpson**	**chao1**
S3_SS4	433.00	4.85	0.86	530.06
S4_SS4	358.00	5.82	0.94	394.69
S5_SS4	249.00	5.06	0.91	291.00
C6_DD4	313.00	4.13	0.86	374.92
C1_DD4	255.00	3.13	0.75	301.92
C2_DD4	110.00	1.44	0.38	133.38
	346.6666667	5.243342554	0.902378147	405.2503771
	226	2.902177782	0.663358313	270.0726923

### Differential Gene Expression Between the Selection and Control Lines

**FIGURE 3:**
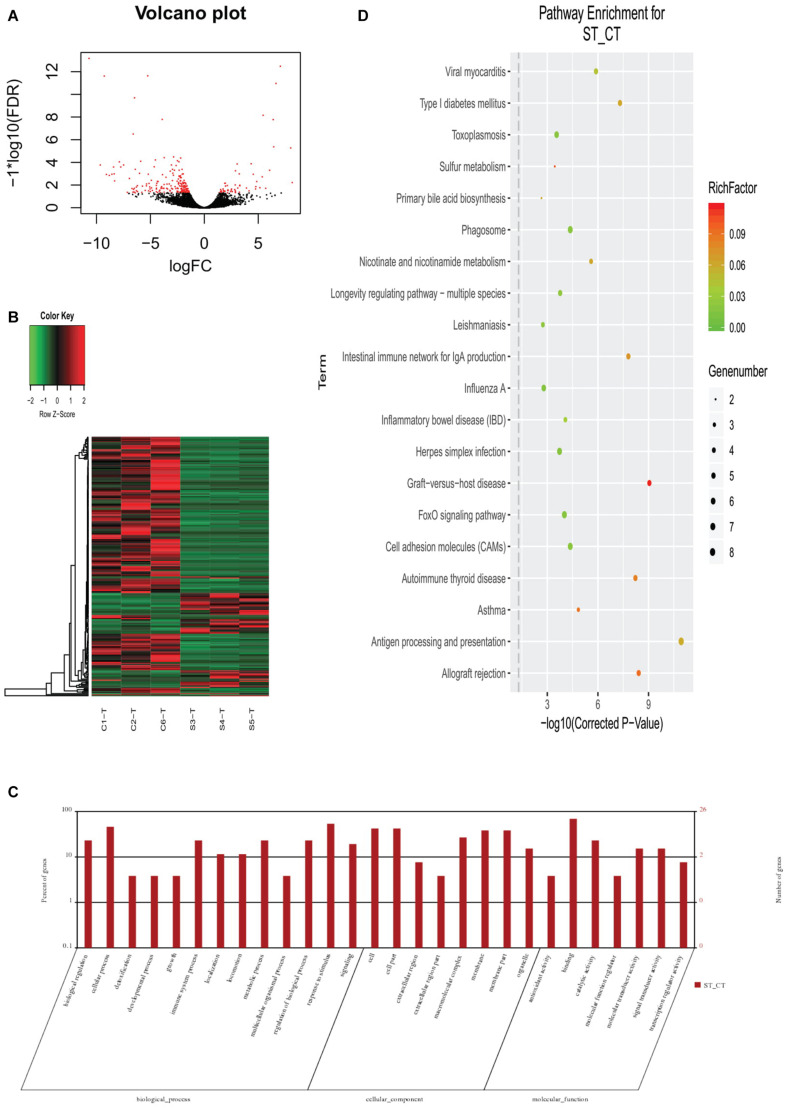
Differentially expressed genes (DEGs) in gut transcriptome between the selection (Huanghe carp new strain 4th generation) and control lines. **(A)** Volcano plot of DEGs. **(B)** Heatmap of DEGs. **(C)** GO functional classification of DEGs. **(D)** KEGG annotation of DEGs. CT means control group, where ST means selection group sampled.

The same samples (as used for the 16S sequences) were used to sequence transcriptomes and identify the DEGs in the selection and control lines of Huanghe carp new strain. In total, an average of 44,670,884 clean reads was obtained per sample from 274,824,364 raw reads for all samples (Supplementary Table 5). The GC content was 47.70% and Q30 was 92.54%. The average mapping rate for all clean reads was 75.70%. Among the 249 significantly DEGs (shown in Figure 3A as a volcano plot and Supplementary Table 6), 194 were downregulated and 55 genes were upregulated (Supplementary Tables 7,8). By GO annotation, these genes were classified into the following functional categories: biological regulation, cellular process, detoxification, developmental process, growth, immune system process, localization, locomotion, metabolic process, multicellular organismal process, regulation of biological process, response to stimulus and signaling (all belong to biological processes); cell, cell part, extracellular region, extracellular region part, macromolecular complex, membrane, membrane part and organelle (cellular components); and antioxidant activity, binding, catalytic activity, molecular function regulator, molecular transducer activity, signal transducer activity, and transcription regulator activity (molecular function). Heatmap analysis showed that selection and control groups had distinct profiles, with DEGs clustered into four parts (Figure 3B). Go annotations for these genes include 13 terms in biological process, eight terms in cellular component and seven terms in molecular function categories (Figure 3C). KEGG annotation assigned most of these genes to the immune-related pathway (Figure 3D).

### Differentially Expressed Genes Related to the Composition of Gut Bacterial Communities

**FIGURE 4:**
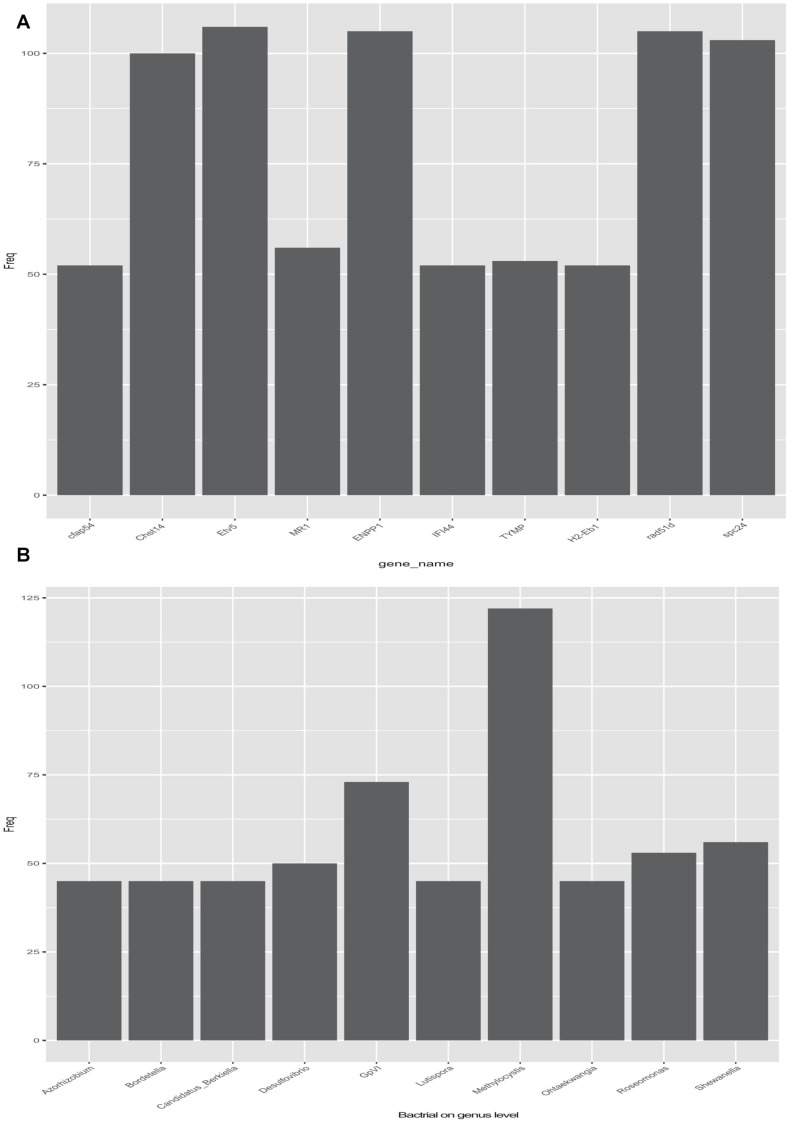
Relationship between differentially expressed genes (DEGs) and bacteria (genus level) in the selection (Huanghe carp new strain 4th generation) and control lines. **(A)** Top 10 DEGs related to gut bacteria. **(B)** Top 10 bacterial genera related with DEGs.

Pearson correlation was used to infer the relationship between the composition of the microbial communities on the genus level and DEGs (Table 2). A total of 2,892 pairs were explored, comprising 245 genes and 256 genera. The top-10 candidate genes with genus-level relationships with the bacterial community are listed in Figure 4A. Many of them were involved in immune responses, such as H-2 class II histocompatibility antigen, E-S beta chain-like (*H2-Eb1*), thymidine phosphorylase-like (*TYMP*), interferon-induced protein 44-like (*IFI44*), and major histocompatibility complex class I-related gene protein-like (*MR1*). Other identified genes had a wide range of functions. Cilia- and flagella-associated protein 54 (*Cfap54*) is required for the assembly and function of cilia and flagella. Carbohydrate sulfotransferase 14-like (*Chst14*) transfers sulfate to the C-4 hydroxyl of *N*-acetylgalactosamine residues in dermatan sulfate. DNA repair protein RAD51 homolog 4 (*Rad51d*), ETS translocation variant 5-like, transcript variant X2 (*Etv5*), and Kinetochore protein Spc24 (*Spc24*) were associated with cell differentiation. The top-10 genera identified by pairing with gene frequencies were: *Methylocystis*, *Ohtaekwangia*, *Roseomonas*, *Shewanella*, *Lutispora*, *GpVI*, *Desulfovibrio*, *Candidatus_Berkiella*, *Bordetella*, and *Azorhizobium* (Figure 4B). Among them, the most abundant was *Methylocystis*, which plays a role in methane cycling.

**TABLE 2:** Relationship between differentially expressed genes and bacteria flora on genus level.

**Gene ID**	**Gene Name**	**Description**	**Chr**	**Gene_biotype**	***p*-value**	***r***	**Genus**
109094505	LOC109094505	insulin receptor substrate 2-like, transcript variant X2	NC_031698.1	protein_coding	0.030271	0.854362	g__Vibrio
109094505	LOC109094505	insulin receptor substrate 2-like, transcript variant X2	NC_031698.1	protein_coding	0.008537	0.92358	g__GpVI
109094505	LOC109094505	insulin receptor substrate 2-like, transcript variant X2	NC_031698.1	protein_coding	0.030271	0.854362	g__Sediminibacterium
109094505	LOC109094505	insulin receptor substrate 2-like, transcript variant X2	NC_031698.1	protein_coding	0.030271	0.854362	g__Ohtaekwangia
109094505	LOC109094505	insulin receptor substrate 2-like, transcript variant X2	NC_031698.1	protein_coding	0.030271	0.854362	g__Zoogloea
109094505	LOC109094505	insulin receptor substrate 2-like, transcript variant X2	NC_031698.1	protein_coding	0.030271	0.854362	g__Azorhizobium
109094505	LOC109094505	insulin receptor substrate 2-like, transcript variant X2	NC_031698.1	protein_coding	0.030271	0.854362	g__Lutispora
109094505	LOC109094505	insulin receptor substrate 2-like, transcript variant X2	NC_031698.1	protein_coding	0.030271	0.854362	g__Bordetella
109094505	LOC109094505	insulin receptor substrate 2-like, transcript variant X2	NC_031698.1	protein_coding	0.030271	0.854362	g__Candidatus_Berkiella
109096216	LOC109096216	uncharacterized LOC109096216	NC_031698.1	lncRNA	0.043294	0.824925	g__GpVI

*This table is very large, so it is available in the attachment. Head 10 lines are supplied here.*

## Discussion

### Relationship Between the Gut Bacterial Community Structure and Growth Performance

In order to assess whether the Huanghe carp new strain’s growth performance is associated with its gut bacterial flora, the selection group and control group were cultured in similar environments during the growth period, and differences in their gut bacterial community structures were explored. At the genus level *Aeromonas* was the dominant taxon in the control group, followed by *Firmicutes* and then *Roseomonas*, while in the selection group, *Roseomonas* was the dominant taxon, followed by *Firmicutes* and then *Aeromonas*. Therefore, the bacterial community structure of these two groups is different. This implies that the host’s (Huanghe carp new strain) genome interacts with the microbiome in a way to select for certain microbial taxa (Levy et al., 2015). In other aspect, the PCA analysis of the bacterial flora revealed that the selection and control groups were clearly distinguishable. Therefore, we can partially estimate the gut bacterial structure of Huanghe carp new strain from its genetic subspecies’ selective microbial community structure character.

Furthermore, the composition of gut bacterial flora can affect the growth performance of common carp. Li et al. (2013) identified the relative abundance of *Firmicutes* over *Bacteroidetes* as a factor contributing to the fast growth of transgenic common carp. These two bacterial taxa were proposed as the “indigenous” flora of the crab (Wang et al., 2019), and we also found that *Firmicutes* comprise a large proportion of the gut flora in the selection group. In humans, the *Firmicutes*/*Bacteroidetes* ratio changes during different life stages. For infants, adults, and elderly individuals, the ratios were 0.4, 10.9, and 0.6, respectively (Mariat et al., 2009). Similarly, with the culture time increasing, the effect of environmental microorganisms on the intestinal microbes of crabs gradually decreased (Wang et al., 2019), so we selected the harvesting time to ensure a relatively stable gut bacterial structure.

### Relationship Between the Gene Expression in the Gut and Growth Performance

Using transcriptome analysis, Zhang et al. (2018) found that only the endocytosis pathway was enriched in DEGs in the 2-month-old cage cultured Huanghe carp new strain (selection) compared to the control group. Similarly, the endocytosis pathway was also significantly differently enriched between control and selection groups in the current study (Supplementary Table 9). The difference between these two studies is that Zhang et al. (2018) identified differentially regulated interleukin-2 receptor subunit beta (*il2rbeta*) in the endocytosis pathway, whereas we identified interleukin-13 receptor subunit alpha-2-like (*IL13RA2*), transcript variant X2, and interleukin-12 subunit beta-like (*IL12B*) in the endocytosis pathway were found in this study. Higher expression of *IL13RA2* supports the intergenerational transmission of metabolic sequelae (obesity/metabolic diseases) of a high-fat diet (HFD) from father to offspring (Ng et al., 2010). This implies that this gene is involved in the diet-effect intergenerational transmission. Similarly, a high-carbohydrate diet has a positive effect on the growth of and polyunsaturated fatty acid content in the Huanghe carp new strain (Zhang et al., 2018). *IL12B* and *IL12A* produce the heterodimer known as interleukin IL-12, which not only elicits intestinal inflammation but also protects the host against microbial invasion (Danese, 2012). It achieves this by polarizing the maturation of T cells to the Thl phenotype that centrally orchestrates the cellular immune response through the release of *IFN-y*, *IL-2*, and lymphotoxin (Locksley, 1993), which may provide a health boost in the Huanghe carp new strain.

### Relationship Between the DEGs and Bacterial Community Composition in the Gut

Generally, gut bacteria interact with the expression of some genes in the gut epithelial cells (Larsson et al., 2012; Broderick et al., 2014; Richards et al., 2019). Larsson et al. (2012) found that MyD88 was essential for microbiota-induced colonic expression of the antimicrobial genes *Reg3*β and *Reg3*γ in the epithelium. The absence of MyD88 caused a shift in bacterial diversity and a greater proportion of segmented filamentous bacteria in the small intestine. In contrast to gene expression in the *Drosophila melanogaster* wild-type, 53% of upregulated genes of the immune-deficient line exerted their effects through the immune deficiency (*Imd*) pathway, and the remaining through stem cell proliferation (*Upd3*, *Socs36e*, and *Pvf1*) and differentiation (multiple Notch pathway components), as well as higher-oxidative-stress genes, lipid metabolism, and endopeptidase activity pathways (Broderick et al., 2014). When human colonic epithelial cells (HCoEpiC) were treated with live gut microbiota extracted from five healthy human individuals, the strongest response (3,240 genes across any of the five microbiota samples) occurred at 2 h; 588 transcript-by-taxon pairs corresponded to 121 host genes with changes in expression associated with the abundance of 46 taxa (Richards et al., 2019). Among them, *Streptococcus* was also found in the present study. Combining the gut bacterial community structure and DEGs in selection and control group analysis, we found that the *Aeromonas* and *Roseomonas* percentage, as well as differential expression of *IL12*, were related to anti-disease ability; and that the percentage of *Firmicutes* was related to growth performance in the Huanghe carp new strain.

Besides the frequency of related bacteria at the genus level, we also focused on contributions of different genes to the growth, immune and higher muscle polyunsaturated fatty acid content performance of Huanghe carp new strain. In total, all these genes could be divided into four classes. The first class is associated with immune ability, as illustrated by 4.2 and 4.3 in the discussion. The second class is growth performance, comprising genes such as insulin receptor substrate 2-like, transcript variant X2 involved in the insulin like growth factor1 signaling pathway (Rui et al., 2001). SPC24 component of NDC80 kinetochore complex mutants exhibit stunted growth, embryo arrest, DNA aneuploidy, and defects in chromosome segregation with a low cell division rate (Shin et al., 2018). These genes may play a key role in the better growth performance of Huanghe carp new strain (Su et al., 2018). The third class is adipocyte differentiation. These genes comprise Krueppel-like factor 13, which acts as a key pro-adipogenic transcription factor via transactivation of PPARγ expression in porcine adipocyte differentiation (Jiang et al., 2015); G0/G1 switch protein 2-like, which directly interacts with adipose triglyceride lipase and mediates G0S2’s inhibitory effects on lipolysis and lipid droplet degradation (Zhang et al., 2017); and the ectonucleotide pyrophosphatase/phosphodiesterase 1 (*ENPP1*) expression level of which is positively related to defective adipocyte maturation (Liang et al., 2007). These genes may be associated with the higher muscle polyunsaturated fatty acid content in this new carp strain (Shengyan et al., 2018). The last class is associated with nerve regulation; e.g., neurexin-1a is strongly associated with neurodevelopmental disorders, so knockout mice exhibited social deficits and increased levels of aggression (Armstrong et al., 2020); Krueppel-like factor 13 inhibits neurite/axon growth in hippocampal neurons partially by inhibiting the cAMP signaling pathway (Ávila-Mendoza et al., 2020). These nerve regulation-related genes may be a reflection of the microbiota-gut-brain axis (Cryan et al., 2019).

These results indicate that gut bacteria can affect the gene expression in the gut, in which the dominant species or bacterial structure may reflect genetic characteristics of the host, i.e., Huanghe carp new strain. Gut bacterial-gene expression profile in the gut can contribute to the health and performance of the host.

## Conclusion

This study presents evidence that gut bacterial communities interact with gene expression. A total of 2,892 pairs (genes and bacteria at genus level) were explored, comprising 245 genes and 256 genera, and most of the observed genes were involved in immunology, the bacterial community, and cell differentiation. The top-10 bacterial genera included *Methylocystis*, *Ohtaekwangia*, *Roseomonas*, *Shewanella*, *Lutispora*, *GpVI*, *Desulfovibrio*, *Candidatus_Berkiella*, *Bordetella*, and *Azorhizobium*. KEGG annotation suggested that most of these genes were located in the immune-related pathways. This result illustrates that the improved growth performance of the new strain of Huanghe carp may be associated with its improved immune responses and their interaction within the gut bacterial structure.

## Data Availability Statement

All of the transcriptome and 16s amplicon sequencing data has been deposited in NCBI under the accession number PRJNA716751. All other datasets generated for this study are included in the article.

## Ethics Statement

The animal study was reviewed and approved by Ministry of Science and Technology of China, 398th file in 2006 (the code for the application or the authorization related to animal management protocols to the Committee of Ethics and Animal Care).

## Author Contributions

SS, PX, and YT conceived the study, contributed to the design of the experiments, acquired funding, and supervised the project. XJ, CZ, YH, ZL, XY, XZ, and JZ performed all the experiments. All authors contributed to the drafting of the manuscript.

## Conflict of Interest

The authors declare that the research was conducted in the absence of any commercial or financial relationships that could be construed as a potential conflict of interest.

## Footnotes

1 http://www.r-project.org/

## Supplementary Material

Supplementary Table 1 Water Quality Parameters.

Supplementary Table 2 Sample Information.

Supplementary Table 3 Clean Data Statistics.

Supplementary Table 4 OTU Table.

Supplementary Table 5 All Filter Statistics.

Supplementary Table 6 ST CT GO2gene.

Supplementary Table 7 DEG2Re.

Supplementary Table 8 ST CT KEGG.

Supplementary Table 9 Alignment Statistics Combined.
